# (A Critical Appraisal of) Classification of Hypereosinophilic Disorders

**DOI:** 10.3389/fmed.2017.00216

**Published:** 2017-12-05

**Authors:** Jean Emmanuel Kahn, Matthieu Groh, Guillaume Lefèvre

**Affiliations:** ^1^Service de Médecine Interne, Centre de Référence des Syndromes Hyperéosinophiliques-CEREO, Hôpital Foch, Université Versailles-Saint Quentin en Yvelines, Suresnes, France; ^2^Service de Médecine Interne, Hôpital Saint Louis, Université Paris-Diderot, Paris, France; ^3^Université de Lille, INSERM, CHU Lille, U995 – LIRIC – Lille Inflammation Research International Center, Institut d’Immunologie, Centre de Référence des Syndromes Hyperéosinophiliques-CEREO, Unité d’Immunologie Clinique, Lille, France

**Keywords:** hypereosinophilic syndrome, hypereosinophilia, classification, eosinophilic granulomatosis with polyangiitis, eosinophilic disorders

## Abstract

Hypereosinophilia (HE) is a heterogeneous condition that can be reported in various (namely inflammatory, allergic, infectious, or neoplastic) diseases with distinct pathophysiological pathways. In 1975, Chusid et al. published the first diagnostic criteria of hypereosinophilic syndromes (HES). Over the years, as both basic and clinical knowledge improved, several updates have been suggested, with a focus on better distinguishing isolated or asymptomatic eosinophilia from diseases with specific eosinophil-related organ damage. Moreover, underlying molecular and cellular mechanisms of eosinophilia gradually became the cornerstone of successive attempts to classify HE-related diseases. In 2011, the International Cooperative Working Group on Eosinophil Disorders criteria emerged from a multidisciplinary Working Conference on Eosinophil Disorders and Syndromes, and provided substantial contribution to the clarification of general concepts and definitions in the field of HE. Yet, owing to the low prevalence of HE/HES, to the numerous diseases encompassed in the spectrum of HE-related disorders (with sometimes overlapping phenotypes), many questions are left unanswered (e.g., the need to better standardize the use of modern molecular tools, or the clinical relevance of distinguishing different subtypes of idiopathic HES). Here, we review the current state of knowledge in the fields of classification and diagnosis criteria of HE-related diseases, with emphasis on the analysis of both strengths and weaknesses of present concepts and their usefulness in daily practice.

## Introduction

The concept of “hypereosinophilic syndromes” (HES) was introduced by Hardy and Anderson in 1968 (1), and Chusid et al. later suggested in 1975 diagnostic criteria for HES (2). These readable and easy-to-use criteria comprised chronic (i.e., longer than 6 months) hypereosinophilia (HE) (i.e., above 1.5 × 10^9^/L) with no identifiable cause, associated with clinical manifestations. Nowadays, given the various identified molecular mechanisms underpinning HE (e.g., T-cell-dependent IL-5 production, clonal abnormalities of the myeloid lineage) and the subsequent heterogeneity of diseases encompassed in the spectrum of HES, this first set of diagnostic criteria has become outdated. Hereafter, we will review the main classifications in HES, and discuss their strengths and potential pitfalls.

## Current Classifications and Definitions of Eosinophilic Disorders

Initially, the concept of HES was commonly applied to patients with multi-organ damage (often involving the heart) and unexplained chronic HE above 1.5 × 10^9^/L. Yet, different clinico-biological phenotypes were already observed in the first published series of patients with HES, suggesting that various underlying pathophysiological processes could be involved (2, 3). Hence, a subgroup of patients presented with features suggestive of myeloproliferative neoplasm (i.e., anemia, splenomegaly, myelofibrosis, etc.), which led to the concept of myeloproliferative (“leukemic” for Chusid) HES. Decades later, clonal abnormalities involving fusion transcripts (among which PDGFRA and PDGFRB genes) were identified in the same subgroup of patients, thereby validating *ex post* the initial hypothesis (4). Next, the lymphoid variant of HES was defined in another subgroup of patients with dermatologic manifestations that responded to corticosteroids and were originally classified as hypersensitivity (or non-malignant or allergic) HES but in which abnormal clonal T-cells (e.g., CD3^−^CD4^+^ T-cells) that produced eosinophilopoietins were later identified (5).

In the early 2000s, numerous expert classifications embedded the above-defined concepts of molecularly defined myeloproliferative-HES, lymphoid HES, and idiopathic HES (when no molecular or immunological abnormality can be found) (6, 7). Yet, less than two decades later, these classifications already seem outdated due to recent major breakthroughs in molecular biology. Currently, the two main—and partially redundant—classifications are the one proposed by the WHO (which covers only primary/neoplastic HES) (8) and the one proposed by the International Cooperative Working Group on Eosinophil Disorders (ICOG-EO) in 2011 (9). The ICOG-EO (an international and multidisciplinary panel of experts) agreed on unifying terminologies and criteria, and suggested a classification that delineates various forms of HE and HES (including primary and secondary variants) based on specific hematologic and immunologic conditions.

### Definitions of Eosinophilia and HE

International Cooperative Working Group on Eosinophil Disorders first provided basic definitions of what should be considered as HE (Table 1). The cut-off of 1.5 × 10^9^/L was chosen to differentiate HE (>1.5 × 10^9^/L) from “eosinophilia” (between 0.5 and 1.5 × 10^9^/L). The duration of 1 month of blood HE (instead of the 6 month delay comprised in Chusid criteria) was retained as sufficient and indeed makes sense from a clinical viewpoint, considering that life-threatening organ involvement is frequent in HES. Importantly, the latter criteria also include tissue eosinophilia in the field of HE-related disorders, thereby highlighting the fact that discrepancies between blood and tissue eosinophilia (e.g., marked tissue eosinophilia without blood eosinophilia or HE) can be reported in some eosinophilic disorders (e.g., eosinophilic esophagitis or acute eosinophilic pneumonia). Hence, the pathologist’s definition of tissue HE includes more than 20% of eosinophils in bone marrow sections, and/or (for other tissues) extensive tissue infiltration by eosinophils, and/or marked deposition of eosinophils granule proteins (Table 1).

**Table 1:** Summary of the ICOG-EO’s definitions of eosinophilic disorders.

Term	Definition and criteria	Subtype
Blood eosinophilia	Eosinophils > 0.5 × 10^9^/L in blood	
Hypereosinophilia	Eosinophils > 1.5 × 10^9^/L in blood on 2 examinations (interval > 1 month) and/or tissue HE defined by the following: 1. Percentage of eosinophils in BM section exceeds 20% of all nucleated cells and/or 2. Pathologist is of the opinion that tissue infiltration by eosinophils is extensive and/or 3. Marked deposition of eosinophil granule proteins is found (in the absence or presence of major tissue infiltration by eosinophils).	HE_FA_ HE_US_ HE_N_ HE_R_
Hypereosinophilic syndrome	1. Criteria for peripheral blood HE fulfilled and 2. Organ damage and/or dysfunction attributable to tissue HE, and 3. Exclusion of other disorders or conditions as major reason for organ damage.	HES_I_ HES_N_ HES_R_
Eosinophil-associated single-organ diseases	1. Criteria of HE fulfilled and 2. Single-organ disease	

*HE, hypereosinophilia; HE_FA_, familial (hereditary) hypereosinophilia; HES, hypereosinophilic syndrome; HE_US_, hypereosinophilia of undetermined significance; HEN, Primary (clonal/neoplastic); HE_R_, secondary (reactive) hypereosinophilia; HES_I_, idiopathic hypereosinophilic syndrome. Adapted from Valent et al. (9)*.

### Definition of HES

The ICOG-EO defined HES as blood HE or tissue HE associated with HE-related organ damage (precluding the absence of an alternative diagnosis for the organ dysfunction) (Table 1). As compared with the Chusid criteria, this definition comprises a causal link between the observed tissue HE and organ damage, the probability of which is considered sufficient in presence of the following specific histological findings: (1) fibrosis or (2) thrombosis or (3) cutaneous eosinophilia (with erythema or angioedema or pruritus or eczema or ulceration) or (4) peripheral or central neurologic defect.

### Definition of Neoplastic HE/HES (HE_N_/SHE_N_)

Hypereosinophilia or HES are considered as neoplastic (or clonal or primary, HE_N_/SHE_N_) when an underlying myeloid/lymphoid/stem cell neoplasm with HE and rearrangement of *PDGFRA, PDGFRB, FGFR* or with *PCM1-JAK2* translocation is identified.

HE_N_/HES_N_ also encompasses the broad spectrum of other WHO-defined myeloid neoplasms with associated eosinophilia, such as *BCR-ABL1*^+^ chronic myeloid leukemia, *JAK2-*mutated myeloproliferative neoplasms, *KIT D816V^+^* systemic mastocytosis, acute myeloid leukemia (AML) associated with CBFβ fusion gene, myelodysplastic syndromes associated with HE, and other WHO-defined myeloid neoplasms with HE (10).

Finally, HE_N_/SHE_N_ also includes a last subgroup of patients classified as chronic eosinophilic leukemia not otherwise specified, which should remain an exclusion diagnosis defined by (1) the exclusion of all genetically predisposed conditions described previously, (2) the absence of molecular or cytological features of AML, and (3) the presence of a non-specific clonal cytogenetic or molecular abnormality (i.e., TET2, ASXL1, IDH2, SF3B1) or blast cells >2% in the blood and >5% in the bone marrow.

### Definition of Reactive HE/HES

Reactive HE and HES (HE_R_ and HES_R_) aggregate all conditions (e.g., parasitic infections, adverse drug reactions, inflammatory, or neoplastic diseases) in which eosinophils are considered as non-clonal and are thought to be driven by Th-2 (mainly IL-5) cytokines. Importantly, although it may seem counterintuitive at first sight, ICOG-EO classification emphasizes the fact that HE_R_ and HES_R_ (with reactive eosinophils) can be observed in neoplastic diseases in which the clonal cells (T-cells, Reed-Steinberg cells, carcinomatous cells, mast-cells, etc.) are the main sources of IL-5 and other eosinophilopoietins. Hence, the lymphocytic variant of HES (an indolent T-cell lymphoproliferative disease) is, therefore, classified as a subtype of HES_R_.

### Definition of Idiopathic HES

When a patient fulfills the criteria of HES but does not comply with the definitions of both HES_N_ and HES_R_, the diagnosis of idiopathic HES (HES_I_) can be retained. Interestingly, in main expert centers in HES, more than half of HES patients are classified as HES_I_, while 10–20% of patients each belong to HES_N_ and HES_R_ categories (11).

### Definition of HE of Undetermined Significance

Patients with isolated blood HE but without organ dysfunction and who will remain completely asymptomatic over time are not that uncommon. Hence, after that an initial comprehensive evaluation excludes HE_N_ and HE_R_, the ICOG-EO classification suggests that such patients should be classified as HE of undetermined significance (HE_US_). This newly defined subgroup has major clinical implications since recent data strongly suggests that such patients carry a good prognosis and should only be closely followed without treatment (12).

## Old and New Criteria for the Classification of Neoplastic HE/HES

In the 2005 and 2010 classifications of HE/HES, experts brought to the forefront “good old fashioned” features suggestive of a myeloid neoplasm (i.e., hepatosplenomegaly, increased serum vitamin B12 or tryptase, anemia, thrombocytopenia, myelofibrosis) as criteria for “myeloproliferative-HES,” even in the absence of a molecularly proven HES_N_ (6, 7). With the exception of the blast cell count, the ICOG-EO and WHO classifications have nearly completely excluded these patients—which are now classified as HES_I_—from the field of HES_N_. This distinction of patients with presumed myeloid neoplasm is clinically relevant as response to different treatment options differs (less response to steroids, more likely to respond to imatinib). Hitherto, such patients carry a guarded prognosis [the latter being closer to HES_N_ than that of other HES_I_ patients (13, 14)] and, as patients with HES_N_, may require treatment with tyrosine kinase (TK) inhibitors, cytotoxic drugs, or even bone marrow transplantation. Hence, the current distinction between patients with molecularly defined HES_N_ from those with similar clinical features but without any identifiable mutation is questionable, and it seems desirable that further updates of HE/HES classifications should individualize these myeloproliferative-HES patients as a specific subgroup even in the absence of an identified mutation.

Next, due to the development of new sequencing methods and tools in malignant hematology [especially the next-generation sequencing (NGS)], the field of neoplastic HES has considerably evolved. Since the *FIP1L1–PDGFRA* fusion transcript gene was discovered in 2003, the list of genetically defined HE has regularly been implemented over the years and now comprises 72 distinct entities consisting mostly of TK fusion genes (10).

The identification of numerous mutations in myeloproliferative disorders and myelodysplastic syndrome raise the question whether these new mutations should be included in further classifications of HES. Two recent studies report NGS results in two cohorts of 98 and 51 patients with HE_US_ and/or HES (15, 16). Interestingly, such mutations (including *ASXL1, TET2, SETBP1, CSFR3*, and SF3B1) were identified in 11 and 28% of patients, respectively, suggesting that a significant proportion of patients otherwise classified as HE_US_ and/or HES_US_ might rather belong to the HE_N_ and HES_N_ subgroups. Yet, none of these studies provided convincing elements demonstrating the transforming capacity of these mutations, suggesting that they may not be the true driving mutation for HES/HE_US_. In addition, the impact of NGS-identified mutations on survival remains an open question, a poorer prognosis in patients with NGS-mutations being suggested in a single study (16). Hence, NGS seems to be a highly powerful tool to identify molecular defects in HE/HES. Yet, large prospective registries are needed in order to evaluate its potential usefulness in daily practice regarding patients’ treatment and prognosis, before this tool be included as a new criterion for HE_N_ or HES_N_.

## Reactive HES: A Disorder with Bystander HE or a True Reactive HES?

Many pathologic conditions can induce reactive blood and/or tissue eosinophilia (HE_R_), due to the overproduction of eosinophilopoietic cytokines, mostly IL-5. Yet, in many of these situations, skin and organ damage seem to be due to an autoimmune process (e.g., bullous pemphigoid), a malignant disease (e.g., cutaneous lymphoma, histiocytosis, mastocytosis) or to the massive tissue infiltration by IgG4^+^ plasma cells (e.g., IgG4-related disorders) rather than to eosinophilic tissue infiltration. From a pathological viewpoint, substantial effort (including assessment of extracellular deposition of eosinophil granule proteins by immunohistostaining) has been made by the ICOG-EO classification to define eosinophilic tissue infiltration. Yet, these laboratory techniques are often neither standardized nor performed routinely, and not used as a diagnostic tool in daily practice.

On the other side, in some patients with solid cancer, lymphoma or helminthiasis, a pronounced blood and tissue eosinophilia may occur in organs other than those affected by the underlying disease. In such situations, a true eosinophilic endomyocardial fibrosis due to eosinophil toxicity—as well as many other organ involvements—have been reported. Hence, physicians must be aware that the same disease may induce a bystander blood and/or tissue HE without meaningful consequences related to eosinophils, or a true reactive HES_R_.

According to the clinical context, the initial workup of an unexplained HE/HES must include broad investigations in order to rule out with certainty an underlying disease likely to favor HE_R/_HES_R_. The choice of keeping HES_R_ as part of the ICOG-EO’s nosology has clinical implications: (1) HES_I_ is an exclusion diagnosis which supposes that all etiologies of HES_R_ must be excluded and (2) the treatment of the underlying cause may reverse HE_R_, but in case of organ damage and/or dysfunction attributable to tissue HE (i.e., HES_R_), corticosteroids may be considered from the outset in addition to the treatment of the underlying disease.

## Unmet Needs in the Diagnosis of the Lymphoid Variant of HES

HES_L_, a subtype of HES_R_, is a chronic clonal indolent T-cell lymphoproliferative disorder in which mature peripheral T-cells secrete high amounts of IL-5, leading to the polyclonal expansion of eosinophils. Hence, to some extent, HES_L_ can be considered as the archetype of Th-2 driven eosinophilic disorders (17). Patients can be aymptomatic or poorly symptomatic for years, with HE being the sole manifestation of the indolent T-cell proliferative disorder (18). Diagnosing HES_L_ is important for three reasons: (1) its treatment can differ from that of HES_I_, notably because of frequent corticosteroid dependency requiring additional corticosteroid-sparing-treatments, (2) clonal T-cells that are found in blood and tissues of HES_L_ patients can be mistaken for aggressive T-cell lymphoma, and patients wrongly treated as such with chemotherapy, and (3) authentic T-cell lymphomas (e.g., angioimmunoblastic T-cell lymphomas) may occur during disease course of these patients, who should be closely monitored (18–20).

Diagnosing HES_L_ usually requires the detection of an abnormal T-cell subset in the peripheral blood. Experts agree that three main subsets of HES_L_ must be systematically searched by flow cytometry: CD3^−^CD4^+^TCRab^−^, CD3^+^CD4^+^CD7^−^, and CD3^+^CD4^−^CD8^−^TCRab^+^ (21). Although lacking specificity, further confirmation of HES_L_ is supported by a clonal TCR rearrangement.

Yet, a clear definition of what should (or should not) be diagnosed as HES_L_ is lacking in all current classifications. Pertinently, should a cutoff of absolute or relative counts of such abnormal T-cells be defined? Is the demonstration of their ability to produce high levels of IL-5 necessary? Is a clonal TCR rearrangement necessary or sufficient to define HES_L_? Could other abnormal subsets of blood cells (e.g., type 2 innate lymphoid cells) define HES_L_? Last, several biological biomarkers such as IL-5, CCL-17/TARC, IgE, which demonstrated in many studies potential utility in their ability to distinguish between various subtypes of HES (18, 22) could also be added as additional diagnostic criteria in future classifications.

## Idiopathic HES: A Multifaceted Disease with Many Overlapping Conditions

### Should Multi-Organ and Single-Organ Diseases Be Distinguished?

According to current definitions, all patients with blood HE and organ damage with significant eosinophilic infiltration could be classified as HES, whatever the number of organs involved.

Yet, the ICOG-EO classification also makes a clear distinction between HES and several other organ-restricted conditions with HE (e.g., eosinophilic cystitis, eosinophilic esophagitis, eosinophilic gastroenteritis, eosinophilic pneumonia, dermatologic conditions associated with HE, etc.) that by definition only affect a single organ during the entire course of the disease (the main suggested reason being that the causative role of eosinophils in organ damage is unclear) (9). Nevertheless, some patients with HES_L_, *FIP1L1–PDGFRA* HES_N_ or HES_I_ may only have single-organ involvement at disease onset, with further organ involvement only occurring during follow-up (22). Furthermore, the single-organ damage also depends on the way the clinician looks at the patient: in chronic eosinophilic pneumonia, many patients have sinonasal polyposis. Should they be classified as single-organ disease or HES? Hence, such separation at diagnosis between eosinophilic single-organ diseases and HES appears questionable. In our mind, most patients with eosinophilic single-organ disorders demonstrated by histopathology should be investigated and subsequently followed as HES, even though some of these patients will indeed subsequently never develop multi-organ HES.

### Should Outcome Profiles of HE_I_/HES_I_ Be Distinguished?

**Figure 1:**
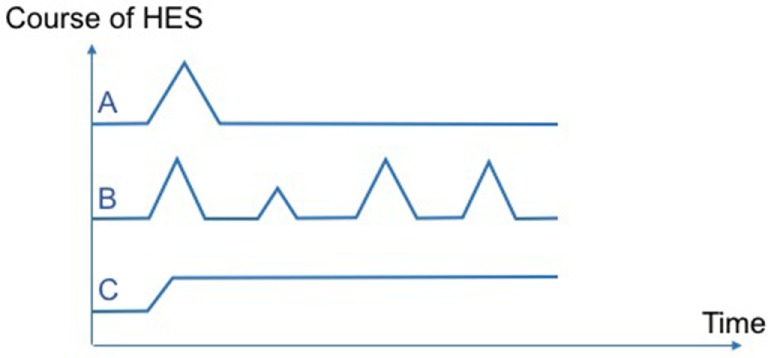
Various patterns of disease courses observed in hypereosinophilic syndromes (HES). Pattern **(A)**: single flare without subsequent relapse. Pattern **(B)**: several relapses with intervals of complete remission. Pattern **(C)**: chronic persistent disease.

In daily practice, HE_I_/HES_I_ treating physician are confronted with various patterns of disease courses. First, some patients will present a single flare of variable duration and will recover (spontaneously or after corticosteroids tapering and discontinuation) without subsequent relapse (Figure 1A). Of note, according to previous classifications of HES disorders (6, 7), these latter patients with less than 6 months of disease duration would not have been classified as HES *per se*. Next, other patients periodically relapse during follow-up, with a variable delay in between flares (that can be weeks, months or years in some cases, and whose severity may also fluctuate overtime). Hence, a pragmatic approach to treating such patients could be short courses of corticosteroids but without long-term therapy (Figure 1B). Notably, both outcome profiles have been reported in various single-organ eosinophil disorders [e.g., acute eosinophilic pneumonia, eosinophilic gastroenteritis (23), episodic angioedema with HE (Gleich’s syndrome) (24)] as well as in HES_I_. Last, a third set of patients will present, usually in the context of corticosteroid dependency, chronic persistent disease requiring second-line treatments (Figure 1C).

Underlying mechanisms involved in eosinophilia are likely to differ between the three disease patterns described here above. Hence, a single flare of HES without subsequent relapse (pattern A) strongly suggests temporary exposure to an extrinsic trigger (e.g., drug-induced eosinophilia for the disease). Conversely, recurrent or chronic persistent HE_I_/HES_I_ (patterns B and C) suggest an intrinsic dysfunction of eosinophil regulation and/or a persistent unidentified underlying cause. By analogy with multiple sclerosis, it is advisable that these different patterns of disease courses be incorporated into further updates of disease classifications in an effort to homogenize inclusion criteria in clinical trials and to individualize patient care.

### Antineutrophil Cytoplasm Antibody (ANCA)-Negative Eosinophil Granulomatosis with Polyangiitis (EGPA) and HES_I_: The Diagnostic (and Therapeutic) Dilemma

Eosinophil granulomatosis with polyangiitis is defined in the 2012 International Chapel Hill Consensus Conference on the Nomenclature of Vasculitides as an eosinophil-rich and necrotizing granulomatous inflammation often involving the respiratory tract, with necrotizing vasculitis predominantly affecting small to medium vessels, and associated with asthma and eosinophilia (25). EGPA differs from other ANCA-associated vasculitides (AAV) by the constant presence of asthma, blood and tissue eosinophilia, and the low prevalence of ANCA positivity, which are detected in only 20–40% of patients (26). Next, the phenotype of ANCA-positive and ANCA-negative EGPA patients differ, with ANCA-negative patients having less vasculitic manifestations (purpura, peripheral neuropathy, glomerulonephritis, scleritis) but more frequent cardiomyopathy (often mimicking that observed in HES) (27). Considering that ≈40% of patients with asthma, HE above 1.5 G/L and at least another systemic manifestation had neither symptoms of vasculitis nor ANCA, a recent European Respiratory Society-endorsed Taskforce on EGPA suggested that this subgroup of patients be considered to have hypereosinophilic asthma with systemic (non-vasculitic) manifestations (HASM) rather than genuine EGPA (28). The results of a negative trial testing azathioprine versus placebo in low-risk EGPA (29) and the recent MIRRA study demonstrating the benefit of mepolizumab in EGPA (30) both confirm the overlap between ANCA-negative EGPA and HES. Given the fact that clinical and biological profiles of ANCA-negative EGPA and HES overlap markedly (31), it seems appropriate to consider a diagnosis of HES in patients with HASM should be mentioned in further updates of both AAV and HES classifications (32).

## Conclusion

The concept of HES has evolved considerably since the first classification by Chusid in 1975, and the recent ICOG-EO classification has successfully embedded most of the field’s recent breakthroughs. These include, albeit not exclusively, the identification of numerous TK fusion genes, the concept of HES_R_ (among which HES_L_), and the need for a clear histopathological definition of eosinophilic tissue infiltration. Moreover, this classification puts an end to many longstanding issues in the HE/HES domain and is a useful tool for the physician in daily care, allowing for better classification of patients between single-organ disease, HES_N_, HES_R_, HES_I_, and HE_US_ (a condition that does not require therapy). Nevertheless, due to the lack of large prospective cohorts of HE/HES patients, one major limitation of the ICOG-EO classification is that it is mainly expert based and, thus, remains low-evidenced. Pertinently, many points (e.g., the need for a clear definition of HES_L_; how to treat patients with a myeloproliferative phenotype but for whom a clonal mutation is not (yet) evidenced; improving the diagnostic workup of patients suspected of ANCA-negative EGPA versus HES_I_) are open for improvement and should be the starting point of future HE/HES-targeted research. Hence, the implementation of international multicentric registries is awaited in order to improve current classifications of HE/HE and subsequently patient care.

## Author Contributions

All authors listed have made a substantial, direct, and intellectual contribution to the work and approved it for publication.

## Conflict of Interest Statement

The authors declare that the research was conducted in the absence of any commercial or financial relationship that could be construed as a potential conflict of interest. The handling editor declared a past supervisory role with one of the authors GL.
